# Salivary Proteins in Human Acquired Enamel Pellicle (AEP) on Eroded and Uneroded Teeth in Patients with Gastro-Oesophageal Reflux Disease (GORD)

**DOI:** 10.3390/dj12080235

**Published:** 2024-07-26

**Authors:** Rasha Alharthi, Mahdi Mutahar, David Bartlett, Jafar Jafari, Rebecca Moazzez

**Affiliations:** 1 Department of Clinical Dental Sciences, College of Dentistry, Princess Nourah bint Abdulrahman University, Riyadh 11671, Saudi Arabia; 2 School of Dentistry, Health and Care Professions, University of Portsmouth, Portsmouth PO1 2QG, UK; 3 Centre for Oral Clinical and Translational Sciences, King’s College London, London SE1 9RT, UK; david.bartlett@kcl.ac.uk; 4 Upper GI Physiology Service, Guy’s and St. Thomas’ Hospitals NHS Foundation Trust, London SE1 9RT, UK; jafar.jafari@gstt.nhs.uk; 5 Department of Preventive and Restorative Dentistry, University of the Pacific, San Francisco, CA 94103, USA; rmoazzez@pacific.edu

**Keywords:** erosion, tooth wear, saliva, enamel, AEP, GORD

## Abstract

The aim of this in vivo study was to compare total protein present in the salivary films (F) and acquired enamel pellicle (AEP) on eroded and non-eroded surfaces in patients suffering from GORD symptoms with and without GORD diagnosis (GORD, No-GORD). Thirty-nine patients suffering from GORD symptoms and erosive tooth wear on lower first molars and an unaffected posterior occlusal surface in the same quadrant were recruited from Guy’s hospital, London. Salivary film and AEP were collected from the eroded and uneroded occlusal surfaces, using 0.5% sodium dodecyl sulphate (SDS)-soaked filter papers. Total protein concentration was analysed using bicinchoninic acid assay (BCA). Statistical analysis was conducted using Shapiro–Wilk, ANOVA, and Tukey’s tests (*p* < 0.05), comparing four GDS sample types and GORD vs. No-GORD groups. The level of significance was set as *p* < 0.05. Data were compared between eroded and uneroded surfaces in the same patient with GORD symptoms, as well as between those with or without a GORD diagnosis (GORD, No-GORD). The AEP total protein concentration from the eroded [2.17 (0.49) mg/mL] and uneroded surfaces [2.24 (0.66) mg/mL] of the GORD group were statistically significantly lower than those on eroded [3.27 (1.01) mg/mL] and uneroded [3.33 (1.57) mg/mL] surfaces in the No-GORD group (*p* = 0.007) (*p* = 0.008), respectively. No statistically significant differences were observed for film and AEP between eroded and uneroded surfaces (*p* > 0.05).

## 1. Introduction

Erosive tooth wear (ETW) involves the loss of hard tooth substances due to acid exposure to exogenous (food and drink) and endogenous (stomach acid) sources [1,2,3]. The association between Gastro-Oesophageal Reflux Disease (GORD) and ETW has previously been reported [4,5]. One of the factors that could contribute to the association is the volume or pH of saliva, which can help identify the risk factors for tooth wear and in recommendations for adequate preventive measures to patients [6,7,8]. Saliva is one of the major biological factors protecting both the gastrointestinal tract and the oral system. 

In the oral system, an organic, bacteria-free layer is formed on enamel surfaces as a result of salivary protein adsorption forming an “Acquired Enamel Pellicle (AEP)” and is composed mainly of proteins and glycoproteins [9,10]. Extensive research has demonstrated various protective effects of AEP. AEP acts as a semi-permeable membrane, protecting tooth surfaces, regulating demineralisation and remineralisation, aiding protein adherence, and providing lubrication for better speech and chewing [10,11,12,13]. However, there are limited reports on the effect of in vivo AEP against intrinsic acidic challenges [14]. Wang et al. [15] have highlighted the diagnostic potential of salivary pepsin as a marker for distinguishing sub-types of GORD and related disorders, underscoring the importance of saliva in assessing oral and gastrointestinal health [15]. These findings suggest that salivary components, including proteins found in AEP, could serve as valuable biomarkers in understanding the pathophysiology of ETW in patients with GORD. 

In vitro studies have investigated the protein components of AEP and demonstrated the protection against ETW [11,16,17,18]. However, the unique nature of AEP formed in vivo is different from the in vitro formed AEP for many reasons; there are dissimilarities in the salivary flow rate, dynamics, AEP thickness, mineral components, and enzymatic activities [19,20]. Previous studies by our group have assessed the protective effect of in vivo AEP. Moazzez et al. (2014) reported significant differences in surface roughness (Sa) on the enamel, suggesting that AEP was protective from acidic challenges in healthy individuals compared to those with ETW [21]. Mutahar et al. (2017) reported reduced total protein concentration on in vivo formed AEP of eroded enamel surfaces compared to uneroded enamel surfaces from the same patients presenting with ETW due to dietary acids [22]. 

Intrinsic acids and enzymes are more destructive to teeth than dietary acids [23,24]. GORD is a symptom-driven disease; however, its symptoms are not always linked to diagnosis or changes in the oesophageal lining. Therefore, comprehensive and multidisciplinary management, especially in optimising medication and patient education, is essential for effectively managing GORD [25,26]. A recent study reported that ETW was not correlated to histopathologically diagnosed oesophagitis; hence, patients with non-erosive reflux disease could also develop ETW [27]. However, other recent studies have identified protein differences in AEP and stimulated saliva in patients with GORD, with and without ETW [14,28]. 

This study aimed to compare the total protein concentration of in vivo AEP between teeth with and without ETW in the same patients suffering from GORD symptoms and, in addition, to compare the total protein concentration of in vivo AEP between teeth with and without ETW in patients with and without GORD diagnosis. We hypothesised that the total protein concentration of in vivo acquired enamel pellicle (AEP) differs significantly between teeth with and without erosive tooth wear (ETW) in patients with gastro-oesophageal reflux disease (GORD) symptoms, and between patients with and without a GORD diagnosis.

## 2. Materials and Methods

### 2.1. Ethical Approval and Recruitment

This study was approved by the National Research Ethics Service (NRES) in North East York Research Ethics Committee (REC Ref 18/NE/0099). A power calculation based on published data from patients presenting with ETW as a result of dietary acids [20] and others on protein levels [29,30] using a paired *t*-test and an effect size of 0.6 and 80% statistical power gave a total of 24 participants required to identify a 5% difference at the 5% significance level. A total of 39 participants were recruited accounting for potential dropouts and also due to the availability and willingness of participants. Additionally, this number was chosen to give statistical power to detect a standardised mean difference between participants with GORD symptoms (GDS), patients who were diagnosed with GORD (GD), and those not diagnosed with GORD (NGD).

Thirty-nine patients suffering from GORD symptoms presenting to Guy’s Hospital London for an intraluminal 24 h pH-impedance monitoring test were recruited for this study between April 2018 and November 2019, with a mean age of 49.9 (SD = 16.1), and 21 females and 18 males. Following informed written consent, dental and medical histories were checked and a Basic Erosive Wear Examination (BEWE) was used to assess erosive tooth wear. The inclusion criteria were as follows: aged 18 to 95 years, a minimum of 20 natural occluding teeth, good general health other than GORD symptoms, had a BEWE accumulative score of 12 or more, with at least one score of 3, and had an eroded and uneroded tooth on the same side. Patients were excluded if pregnant or breastfeeding, had severe periodontal disease or active caries on more than one tooth, were unable to speak or understand English, were wearing an appliance, had restoration of the occlusal or incisal surfaces of upper anterior teeth and first molars, had no signs or symptoms of GORD, or did not have an eroded and uneroded surface on the same side.

**Figure 1:**
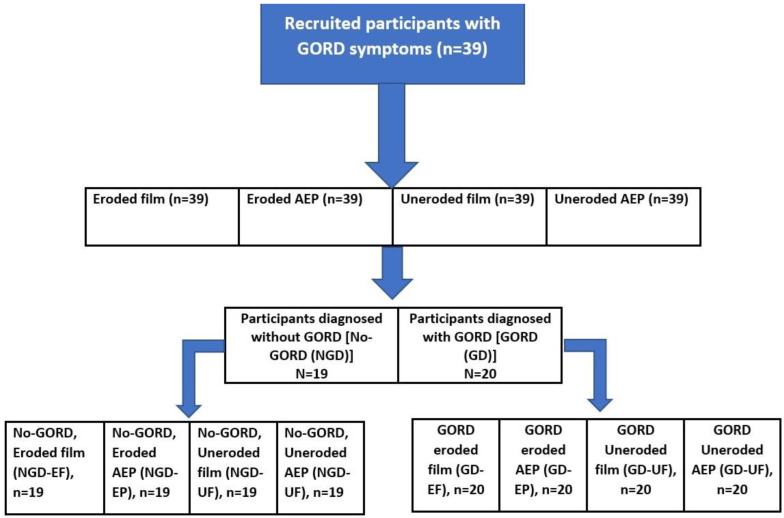
Allocation of groups and samples of salivary films (F) and AEP from eroded (E) and uneroded (U) teeth surfaces in patients diagnosed with GORD symptoms (GD, n = 20) and without GORD symptoms (NGD, n = 19).

The clinical examination was conducted by a single trained and calibrated investigator who performed all oral assessments with the patient in a reclined position and with good lighting. The intra-examiner kappa value was 0.80. For sample collection and analysis, the 39 recruited patients were divided into two main groups as shown in Figure 1. 

Figure 1 shows the number of the recruited participants (n = 39) and the types of samples collected. Thirty-nine participants with symptoms of gastro-oesophageal reflux disease (GORD) were recruited for the study. Salivary film and acquired enamel pellicle (AEP) samples were collected from all participants. After the samples were collected, all participants were screened to determine if they fitted the GORD diagnosis. The diagnosis was based on the GI physiologist’s diagnosis using the intraluminal 24 h pH-impedance [27]. The GORD symptoms assessed were classified according to the global classification of GORD symptoms established by the Montreal consensus, which incorporates evidence from 18 countries. These symptoms include heartburn, regurgitation, chest pain, epigastric pain, dysphagia, belching, and pharyngitis. Twenty participants were diagnosed with GORD. This led to the formation of two subgroups: the first subgroup included 20 patients who were diagnosed with GORD (GD), and the second subgroup consisted of 19 participants who had symptoms but were not diagnosed with GORD (No-GORD, NGD). The mean (SD) of BEWE score of those with GORD (GD) was 15.3 (0.74) and of those for No-GORD (NGD) was 12.8 (0.83) but lacked statistical significance (*p* > 0.05).

### 2.2. In Vivo Sample Collection

Salivary film (F) followed by AEP was collected from each participant (n = 39). The salivary film (F) refers to the initial layer of saliva that forms on oral surfaces, which is subsequently followed by AEP.

**Figure 2:**
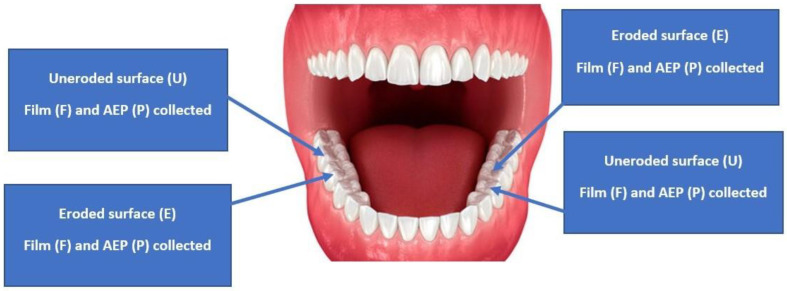
Salivary film and AEP samples were collected from four surfaces in each patient, one eroded and one uneroded adjacent posterior occlusal surface in each of the lower left and right sextants.

Seventy-eight salivary film samples were firstly collected from eroded occlusal surfaces of the lower first molars and seventy-eight salivary film samples from non-eroded adjacent posterior occlusal surfaces (second molars or second premolars). Salivary film samples were collected from four surfaces in each patient by drying the surface with a sialopaper strip, from one eroded and one uneroded adjacent posterior occlusal surface in each of the lower left and right sextants, as shown in Figure 2. The two eroded samples obtained from the same patient were pooled to be analysed together, producing a total of 39 eroded salivary samples (EF). The two uneroded salivary film samples from each patient were also pooled to be analysed together, producing a total of 39 uneroded salivary film samples (UF). The same number of AEP samples were collected and pooled [39 AEP samples from eroded (EP) and 39 AEP samples from uneroded (UP)]. This led to four types of samples being collected: eroded film (EF), eroded AEP (EP), uneroded film (UF), and uneroded AEP (UP). Considering the two subgroups [GORD (GD) and No-GORD, (NGD)], four types of samples were produced from each subgroup, as shown in Figure 1 and Figure 2. 

The salivary film and AEP samples were collected after 12 h of fasting. One sialopaper strip was used for the collection of the salivary film first, and then a new sialopaper strip was used for the AEP collection. The salivary films were collected by applying a dry sialopaper strip, using a sterilised blunt-ended tweezer, for 5 s onto the occlusal surface of the selected, isolated tooth and then placed individually in a microtube [19,20]. AEP was harvested by soaking 5 mm of the sialopaper strip in sodium dodecyl sulphate (SDS) buffer (0.5% *w*/*v*) (Novex, Thermo Fisher Scientific Inc., Loughborough, UK). The solution was prepared by adding 0.5 g of SDS powder using an electronic analytical scale (Fisher Scientific, Loughborough, UK) to 100 mL of deionised water, and stirred using a magnetic stirrer until the SDS particles were dissolved completely in the deionised water. A new SDS solution was made fresh every morning. The sialostrips were placed against the tooth surface to collect AEP and gently rubbed against the occlusal surface for 15 s (approximately 3 × 3 surface area). The AEP samples were placed individually in microtubes, placed on an ice pack, and transported to a freezer where they were frozen at −80 °C until analysis. 

### 2.3. In Vitro Harvesting of AEP Samples

Filter papers carrying the AEP were microcentrifuged and the adsorbed proteins were recovered by adding 15 μL of 0.5% SDS, (1:4) 5 μL of lithium dodecyl sulphate (LDS) buffer and (1:10) LDS buffer (1:4) (Novex, Thermo Fisher Scientific Inc., Loughborough, UK). The AEP eluent underwent microcentrifugation at 8000 rpm for 8 min, followed by the addition of a reducing agent, dithiothreitol (DTT) (1.8 μL, 0.5 mM), at a 1:10 ratio (Sigma-Aldrich, Poole, UK). The samples were then vortexed for 1 min and heat-denatured at 100 °C for 5 min.

### 2.4. In Vitro Experiments

Total protein concentration analysis was carried out using bicinchoninic acid assay (BCA) with purified bovine serum albumin standard (BSA) in a concentration of 2 mg/mL (Pierce Chemical, Rockford, IL, USA) prepared in 96-well plates. A spectrophotometer was used to measure the absorbance of all samples at a wavelength of 562 nm (BioRad laboratories Ltd., Hemel Hempstead, Hertfordshire, UK). The BCA assay has demonstrated high linearity across the tested protein concentrations (R^2^ > 0.98), with accuracy and precision within ICH guidelines [31,32]. It can also detect and quantify low protein concentrations, and the microplate setup allows for large sample screening. However, limitations include interference from agents like lipids and protein-to-protein variability, which can cause over- or under-estimation of the analyte–protein concentration.

### 2.5. Statistical Analysis

Statistical analysis was performed using IBM SPSS Statistics version 23.0 (IBM Corporation, Armonk, NY, USA). Normality was assessed with the Shapiro–Wilk test, and normally distributed data are reported as mean and standard deviation (SD). One-way ANOVA and Tukey’s multiple comparison tests were used, with significance set at *p* < 0.05. Analyses compared four sample types within the GDS group and GORD vs. No-GORD groups. 

## 3. Results

### 3.1. Film and AEP from GORD Symptoms Group (GDS)

Table 1 shows the mean (SD) total protein concentration of in vivo salivary film (F) and AEP (P) from eroded (E) and uneroded (U) surfaces in 39 participants. For the film (F), the mean (SD) of total protein concentration from eroded surfaces was [EF: 2.33 (0.94) mg/mL], and that for uneroded surfaces was [UF: 2.62 (1.59) mg/mL]. For the AEP, the mean (SD) of total protein concentration from eroded surfaces was [EP: 2.74 (0.97) mg/mL], and that for uneroded surfaces was [UP: 2.80 (1.32) mg/mL]. No statistically significant differences were observed for film and AEP between surfaces (*p* > 0.05).

Table 1 presents the total protein concentration (mg/mL) measured in salivary films (F) and AEP (P) from eroded (E) and uneroded (U) surfaces in 39 patients with GORD symptoms (GDS, n = 39). The analysis revealed no significant differences in protein concentration between the groups.

### 3.2. Film and AEP from GD and NGD Groups

Table 2 illustrates the mean (SD) of total protein concentrations in salivary films (F) and AEP (P) from eroded (E) and uneroded (U) surfaces in GORD (GD, n = 20) and No-GORD (NGD, n = 19) patients.

Table 2 shows the total protein concentration (mg/mL) in salivary films (F) and acquired enamel pellicle (AEP) from eroded (E) and uneroded (U) surfaces in GORD (GD, n = 20) and No-GORD (NGD, n = 19) patients. The study included 20 patients diagnosed with GORD and 19 patients with GORD symptoms but without GORD diagnosis. The letters next to the numbers in Table 2 in the last column indicate statistically significant results. Identical letters suggest a statistically significant difference between those groups.

### 3.3. Salivary Film Samples

In the GORD (GD) group, the mean total protein concentration was 1.97 mg/mL for eroded teeth (GD-EF) and 2.22 mg/mL for uneroded teeth (GD-UF). In the No-GORD (NGD) group, the mean total protein concentration was 2.63 mg/mL for eroded teeth (NGD-EF) and 2.97 mg/mL for uneroded teeth (NGD-UF). Although protein concentrations were lower in GD patients compared to NGD patients, there was no statistically significant difference (*p* < 0.05) between the groups.

### 3.4. AEP Samples

For the GORD (GD) group, the mean (SD) of total protein concentration in AEP from eroded sites (GD-EP) was [2.17 (0.49) mg/mL] and that from uneroded sites (GD-UP) was (2.24 (0.66) mg/mL). For the No-GORD (NGD) group, the mean (SD) of total protein concentration in AEP from eroded surfaces (NG-EP) was [3.27 (1.01) mg/mL] while that from the uneroded surfaces (NGD-UP) was [3.33 (1.57) mg/mL].

When comparing the total protein concentration in AEP between eroded and uneroded surfaces within each group [GORD (GD) or No-GORD (NGD) group], there were no statistically significant differences. However, when comparing the AEP total protein concentration between the eroded and uneroded surfaces between the GORD and No-GORD group, statistically significant differences were found. The total protein concentration in AEP from the eroded surfaces of the GORD (GD-EP) group was statistically significantly lower than that of eroded surfaces in the No-GORD (NGD-EP) group (*p* = 0.007). Likewise, the AEP total protein concentration from uneroded surfaces in the GD-UP group was statistically significantly lower than that on uneroded surfaces in the NGD-UP group (*p* = 0.008).

## 4. Discussion

To the authors’ knowledge, this is the first study comparing the total protein concentration of salivary film and AEP on eroded and uneroded surfaces within the same patient with and without symptoms of GORD as well as between patients diagnosed with GORD and those who only show symptoms without a positive diagnosis. There were no statistically significant differences between the total protein concentration on eroded and uneroded surfaces collected from the same patient with symptoms of GORD. However, when AEP was compared on eroded and uneroded surfaces between patients diagnosed with GORD and those without, the total protein concentrations were statistically significantly lower in those with GORD. We used the bicinchoninic acid (BCA) assay with purified bovine serum albumin (BSA) as a standard to measure protein concentration. This method is known for its sensitivity and compatibility with various samples. However, potential variability due to the assay’s limitations, such as interference from substances in saliva, is acknowledged. To improve accuracy, we recommend using more advanced techniques, such as the Pierce™ Quantitative Peptide Assays & Standards, which offer better sensitivity and specificity for peptide quantification, potentially providing more reliable results than traditional assays like BCA.

AEP offers a level of protection to the enamel surface from dietary and oesophageal acids and is a modulator of dental erosion and erosive tooth wear progression [14,23,33]. Hannig [34] examined AEP formed between 1 h and 24 h in an in vitro study and reported that the thickest pellicle was found in 24 h formed AEP with a dense globular layer of 1000 to 1300 nm. It was concluded that the longer the formation time, the thicker the AEP. The collection of film/AEP followed a recently published methodology by our group for dietary erosion participants [22]. Patients were asked to fast for 12 h allowing the formation of AEP. Given the importance of the basal layer for the pellicle’s erosion protection, the method used in this study involved drying the tooth surfaces with a sialopaper strip to collect salivary films, followed by collecting AEP. This is in order to ensure the removal of the basal layer of AEP by gently rubbing 0.5% SDS-soaked sialopaper strips against the tooth surface for 15 s. This approach is well-established for effectively removing adsorbed proteins from the enamel surface while preserving their separation for subsequent identification via two-dimensional gel electrophoresis (2-DE) in future studies [35].

Our results showed significantly lower total protein concentration in AEP from patients diagnosed with GORD compared to those with No-GORD (*p* = 0.007), which could be due to salivary variables. These results suggest that GORD may influence the protein composition of AEP, potentially impacting enamel resilience and susceptibility to erosion.

Research indicates GORD influences changes in salivary mechanisms [34,35,36], impacting both qualitative and quantitative salivary factors associated with its pathogenesis [14,15,28,37,38]. For example, the reduced salivary flow rate and reduced salivary clearance reported in patients with GORD could have resulted in gastric acid remaining longer in the oral cavity compared to in individuals with a normal flow rate [37,39], despite the controversy reported by some other studies that patients with GORD during reflux episodes may show an increased flow rate [40,41].

Martini et al. [28] investigated the proteomic profile of acquired pellicle (AEP) in GORD patients with and without erosive tooth wear (ETW), identifying exclusive membrane proteins in AEP from GORD patients with ETW. In 2023, this research group also analysed proteomic profiles in stimulated saliva among GORD patients, revealing protein alterations. The findings from both studies [14,28] suggest that changes in specific proteins in saliva and AEP may alter the AEP structure, potentially reducing its protective effect and increasing the incidence of ETW. Although our study did not include proteomic analysis, these variations in AEP composition could contribute to the outcomes observed.

Moreover, Hara et al. [42] demonstrated that proteins found in salivary film provided limited protection when formed on dentine. This is particularly relevant since ETW in most GORD patients is severe, often resulting in dentine exposure. They also reported better protection against erosive challenges when the salivary film was formed on enamel surfaces. This might explain our study’s findings, where no significant difference in the total protein concentration of salivary films was observed between eroded and uneroded teeth, as well as between patients with GORD and those without. The results of our study are also in agreement with a previous study by Carpenter et al. [29], who compared the total protein concentration in AEP between thirty patients with and without ETW. Their study reported a lower total protein concentration in AEP of patients with ETW compared to healthy controls. However, Carpenter et al. [29] compared the total protein concentration between healthy individuals and patients diagnosed with dietary erosive tooth wear. In contrast, our study made comparisons between patients diagnosed with and without GORD, with all participants having ETW. This distinction emphasises that our study focused on the presence of GORD as a variable within an ETW-affected population, rather than comparing ETW-affected individuals to healthy controls.

The total protein concentration in film/AEP from GORD patients showed no significant difference between eroded and uneroded surfaces in the same patient. In contrast, Mutahar et al. [22] reported significantly lower total protein concentration on eroded surfaces compared to uneroded surfaces from the same patient diagnosed with dietary erosive tooth wear. This might be because our study included patients with moderate/severe erosive tooth wear (BEWE score of 12 or more with at least one 3), while Mutahar et al. [22] included those with a BEWE score of 8 or more. Another reason could be the continuous acid exposure in GORD compared to the sporadic nature of dietary acid exposure. This affects protein levels due to factors like pH, exposure time, and tooth surface roughness. Hence the protein delivery by the salivary film could be slower compared to those with less severe ETW. This is supported by an in situ study showing no significant difference in the AEP composition on enamel splints and hydroxyapatite between patients with and without ETW [26]. In addition, the AEP maturation time differs from the study by Mutahar et al. [22], in which AEP was collected after an hour of fasting, whereas in our study the AEP collection was done after 12 h of fasting. Therefore, the protein absorption varies in these patients as it depends on many factors such as the pH level, the surface charge, and the surface area. Mutahar et al. [22] investigated the total protein concentration in patients with dietary ETW, whereas our study included patients with intrinsic acid origin. The pH level varies in dietary products ranging between 2.6 and 3.8 [43,44,45], whereas the pH of gastric acid ranges between 0.9 and 1.5 [46], which is more destructive to the dental tissues. The chemical process varies between organic and inorganic acids. Most dietary acids are weak, organic acids causing erosion through a chelation process, whereas gastric (intrinsic) acids are strong, inorganic acids causing erosion through full dissociation in water providing hydrogen ions that dissolve minerals on the tooth surface. This is in agreement with an in vitro study by O’Toole et al. [47] investigating the interaction between enamel, AEP, and extrinsic or intrinsic acids. Their study demonstrated that exposure to citric acid (extrinsic) resulted in a significant decrease in total protein concentration in AEP, whereas exposure to hydrochloric acid (intrinsic) did not lead to significant changes. This highlights the acid-specific effects on AEP composition, which aligns with our study’s observations regarding the presence of unique salivary proteins in GORD patients, irrespective of erosive tooth wear (ETW). These proteins may be linked to the disease itself, suggesting potential biomarkers or targets for understanding and mitigating the oral effects of gastro-oesophageal reflux disease (GORD). In addition, the adsorbed layer of salivary proteins on non-eroded surfaces may modify their adhesive and lubrication properties, altering their tribology and influencing wear and friction. Additionally, the morphology of eroded and uneroded surfaces could affect the desorption or elution of the pellicle, impacting its protective behaviour.

As with many in vivo studies, this research has limitations. Variations in protein amounts may stem from different oral cavity locations (local saliva supply) and challenges in standardising film/AEP collection areas, where larger areas yield more proteins. Another limitation should be noted, that is, because the inclusion criteria required a BEWE cumulative score of 12 or more, with at least one score of 3, this means that dentin was most likely exposed. Therefore, standardisation of whether the AEP was collected from the enamel or dentin surface was not possible in this study, and we could not be certain about the exact surface from which the pellicle was collected. However, the use of standardised sizes of sialostrips does standardise the quantity of proteins harvested from film/AEP samples [46]. In addition, the collected samples were pooled following previous protocols [48,49], and analysis was performed by an investigator blinded to the erosion status of the sample surface in order to increase the amount of collected protein and reduce individual variability, respectively. Future work will include compositional analyses of saliva samples and the role of key salivary proteins against ETW from the study population. In addition, several potential confounding factors, such as variations in diet, oral hygiene practices, and medication use among participants, could influence the results, though further refinement and consideration of these factors are essential for future research. Another point to consider is that despite the statistical insignificance, the groups were different. These differences, even if not statistically significant, must be interpreted with caution as they might suggest underlying variations in the participants that were not accounted for. Further studies with larger sample sizes, better-controlled group characteristics, and the use of more advanced chemicals and instrumentation are necessary to validate our results and provide more definitive insights. 

The findings of this study have important implications for both clinical practice and future research. Clinically, this study suggests that GORD patients have significantly lower total protein concentrations in their AEP, potentially suggesting that these individuals may have compromised enamel resilience and increased susceptibility to erosion. Dental practitioners should be aware of the heightened risk of ETW in GORD patients and consider implementing preventive strategies such as recommending fluoride treatments, dietary modifications, and measures to enhance salivary protection. Additionally, regular monitoring of dental erosion in GORD patients should be a part of routine dental care. Future research should prioritise advanced proteomic analyses, which are needed to identify specific protective proteins in AEP. Larger, longitudinal studies are necessary to validate findings and understand ETW progression. Additionally, examining the effects of dietary and intrinsic acids on AEP composition could inform the development of targeted therapies to enhance enamel protection.

## 5. Conclusions

Our findings indicate that patients diagnosed with GORD exhibit lower total protein concentrations in in vivo AEP compared to those with GORD symptoms only. However, there was no significant difference in total protein concentration between eroded and uneroded surfaces within individuals displaying GORD symptoms. These findings suggest a potential link between GORD and changes in salivary mechanisms, impacting AEP composition and potentially increasing susceptibility to ETW.

## Figures and Tables

**Table 1:** Total protein concentration of in vivo salivary film (F) and AEP (P) from GORD symptoms group (GDS).

Sample Type	Total Protein Concentration (mg/mL) Mean (SD)
Eroded film (EF) (n = 39)	2.33 (0.94)
Uneroded film (UF) (n = 39)	2.62 (1.59)
Eroded pellicle (EP) (n = 39)	2.74 (0.97)
Uneroded pellicle (UP) (n = 39)	2.80 (1.32)

**Table 2:** Total protein concentration of in vivo salivary film (F) and AEP (P) in patients suffering from GORD symptoms (GDS) with and without GORD diagnosis (GD and NGD respectively).

Salivary Film Samples	Total Protein Concentration (mg/mL)	AEP Samples	Total Protein Concentration (mg/mL)
NGD-EF (n = 19)	2.63 (0.84)	NGD-EP (n = 19)	3.27 (1.01)a
NGD-UF (n = 19)	2.97 (2.0)	NGD-UP (n = 19)	3.33 (1.57)b
GD-EF (n = 20)	1.97 (0.92)	GD-EP (n = 20)	2.17 (0.49)a
GD-UF (n = 20)	2.22 (0.81)	GD-UP (n = 20)	2.24 (0.66)b

## Data Availability

The raw data supporting the conclusions of this article will be made available by the authors on request.
